# Comparative Evaluation of Salivary Cathelicidin and 8-Isoprostane Levels Among Smokeless Tobacco Users and Non-users and Their Correlation With Periodontal Health and Disease: A Cross-Sectional Study

**DOI:** 10.7759/cureus.67646

**Published:** 2024-08-23

**Authors:** Mugdha V Karambelkar, Siddhartha Varma, Girish Suragimath, Sameer A Zope, Vaishali S Mashalkar, Apurva V Kale

**Affiliations:** 1 Periodontology, School of Dental Sciences, Krishna Vishwa Vidyapeeth (Deemed to be University), Karad, IND

**Keywords:** smokeless tobacco, periodontitis, isoprostane, cathelicidin, biomarker

## Abstract

Introduction

Periodontal diseases arise from host-microbial interactions influenced by tobacco products. Salivary antimicrobial peptides such as salivary cathelicidin and prostaglandins such as 8-isoprostane are part of the inflammatory cascade affecting periodontal disease pathogenesis.

Methodology

A total of 93 patients, 31 in each group that is healthy, periodontitis, and periodontitis with smokeless tobacco habit patients, were enrolled. The case history was recorded, and clinical examination was performed using periodontal parameter analysis of oral hygiene index simplified (OHIS), Russell’s index, periodontal pocket depth (PPD), and clinical attachment level (CAL). The saliva samples were collected and subjected to an enzyme-linked immunosorbent assay (ELISA) to evaluate cathelicidin and 8-isoprostane. The results were analysed and compared statistically.

Results

The OHIS, Russell’s index, pocket probing depth, and CAL were high in patients with periodontitis and tobacco habit (p<0.001). The cathelicidin levels were the highest in patients with periodontitis and the tobacco habit (1.6 g/mL). The level of 8-isoprostane was the highest in patients with periodontitis with tobacco habit (1.8 pg/mL). Smokeless tobacco users showed higher levels of cathelicidin and 8-isoprostane in periodontitis with tobacco than in the healthy group.

Conclusion

Increased cathelicidin and 8-isoprostane levels in smokeless tobacco users with periodontitis suggest risk biomarkers for tobacco-influenced periodontitis.

## Introduction

The periodontium is a dynamic unit holding the tooth and can lose homeostasis due to inflammation. Periodontal disease is a multifactorial disease with a primary etiology of microorganisms [1]. Dental plaque, as a biofilm, significantly contributes to periodontal disease. Chemical mediators activate the immune response to control the infection [2,3]. However, the exaggerated immune response disrupts homeostasis, leading to tissue destruction. Cathelicidin (LL 37) is a neutrophil-originated antimicrobial peptide. Cathelicidin is released from neutrophils and mononuclear phagocytes. A gradient of cathelicidin surrounds activated leukocytes at a site of infection. It triggers inflammatory reactions, and its concentration can correlate with periodontitis [4]. Isoprostanes are prostaglandin-like compounds formed through free radical-induced peroxidation of arachidonic acid, without enzymes. 8-isoprostane is formed during the inflammatory process, and levels are indicative of the presence of oxidative stress.

Periodontal disease also depends on other factors, such as systemic illnesses, and deleterious habits such as tobacco use. Tobacco products not only cause periodontal destruction but also produce local oxidative stress. Smokeless tobacco affects oral mucosa and periodontal tissues with the direct harmful release of toxic substances and leads to tissue loss in the localised area of application [5]. Smoking negatively impacts the periodontium by reducing blood flow and impairing the immune response, leading to increased susceptibility to periodontal disease. It accelerates the destruction of the supporting bone and connective tissue, resulting in deeper periodontal pockets and tooth loss. Evaluating the effect of smokeless tobacco on inflammatory enzymes and redox reactions is essential to understanding its role in periodontal disease pathogenesis. Saliva can be used as a non-invasive diagnostic biomaterial containing inflammatory proteins, oxidative species, genetic material, etc. Changes in salivary components can indicate disease states [6]. The salivary levels of cathelicidin and 8-isoprostane can be viable biomarkers for evaluating inflammatory conditions [7]. Therefore, this study aims to evaluate and compare the salivary levels of cathelicidin and 8-isoprostane in smokeless tobacco users and non-users, examining their correlation with periodontal health and disease.

## Materials and methods

Material and methods

This cross-sectional study was conducted at the Department of Periodontology, School of Dental Sciences, Karad, following approval from the Ethical Committee of Krishna Vishwa Vidyapeeth (Deemed to be University), Karad (Ethical number: KIMSDU/IEC/08/2022).

Sample size selection

The sample size of 93 was determined using the stratified randomized sampling technique. Ninety-three subjects attending the outpatient section underwent a thorough periodontal assessment. The sample size for the study was arrived at by power analysis. A minimum calculated sample size of 93 (31 samples per group; total of three groups), provided 95% power to detect significant differences and a significance level of 5%.

Inclusion criteria

Patients providing consent for participation in the study were selected. Patients clinically diagnosed with periodontitis according to the Classification of Periodontal and Peri-implant Diseases and Conditions 2017 were included. Patients having a habit of smokeless tobacco such as mishri for a minimum of six years and at least twice daily either by chewing or local placement along with periodontitis were also selected. Patients were divided into three groups after clinical examination.

Exclusion criteria

Patients with a history of systemic disorders; pregnant and lactating females; patients with periodontal therapy within the past three months; patients on antibiotic, antiplatelet, and anti-inflammatory medications; and patients with a habit of smoking, etc. were excluded from the study.

Clinical examination

A total of 93 subjects were chosen and underwent a comprehensive periodontal assessment. Patients were examined clinically based on the simplified oral hygiene index simplified (OHIS) (Green and Vermillon, 1964), Russell’s periodontal index, probing the pocket depth of all teeth, and clinical attachment level (CAL) and divided into three groups with 31 participants in each group: Group A (n=31) with healthy individuals, Group B with patients with periodontitis and no smokeless tobacco habit, and Group C with periodontitis patients with a smokeless tobacco habit.

Sample collection

Saliva was collected from each patient. An unstimulated 2 mL whole saliva sample was collected from each participant between 10.00 AM and 12.00 PM, according to a modification of the method described by Navazesh [8]. The subjects were instructed to avoid eating, drinking, and brushing one hour before sampling. In addition, to avoid contamination of samples with blood, clinical parameters were measured at least one hour before saliva collection. The participants were asked to swallow saliva first and then allow the saliva to drain passively for 5 min over the lower lip into a sterile tube.

Sample storage

Collected samples were centrifuged for 15 minutes at 1,000 rpm at 2-8°C. All the particulates were removed and stored. Collected saliva was immediately stored in an aliquot at <-80°C. Samples were analyzed within two months of collection.

Biomarker analysis

The concentration of salivary cathelicidin and salivary 8-isoprostane was determined with the human enzyme-linked immunosorbent assay (ELISA) kits (ELK Biotechnology Co. Ltd., Wuhan, China) according to the manufacturer’s instructions in the biochemistry laboratory of Krishna Vishwa Vidyapeeth (Deemed to be University), Karad.

Statistical analysis

**Figure 1:**
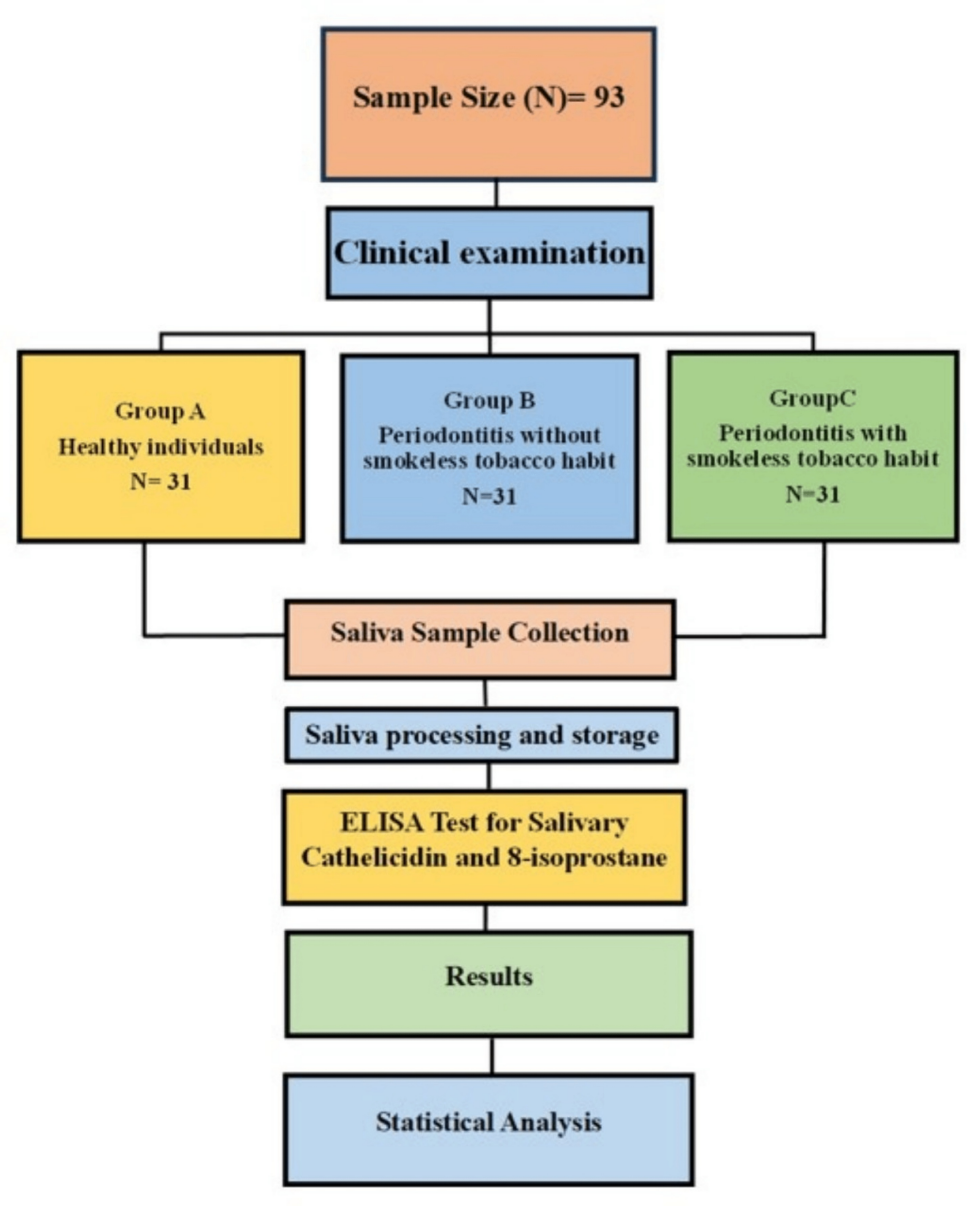
Summary of the study protocol.

The results of biomarker analysis were subjected to statistical analysis using Statistical Product and Service Solutions (SPSS, version 21.0; IBM SPSS Statistics for Windows, Armonk, NY) software. Comparison of various parameters between the groups was done using the analysis of the variance formula test (ANOVA), followed by Tuckey’s post-hoc test. To test whether there was a significant relationship between these parameters, the Pearson correlation coefficient was derived. The level of significance (p-value) was set at p<0.05. The flow chart of the study design is mentioned (Figure 1).

## Results

A total of 93 subjects were included, divided into three groups: the healthy group (Group A, n=31), the periodontitis without smokeless tobacco habit group (Group B, n=31), and the periodontitis with smokeless tobacco habit group (Group C, n=31).

Group A had a mean age of 41.74 years, Group B had 39.29 years, and Group C had the highest mean age at 47.29 years. A total of 60 males and 33 females participated in the study. Among male participants, there were 23 in Group A, 18 in Group B, and 19 in Group C. Female participants were eight in Group A, 13 in Group B, and 12 in Group C.

**Figure 2:**
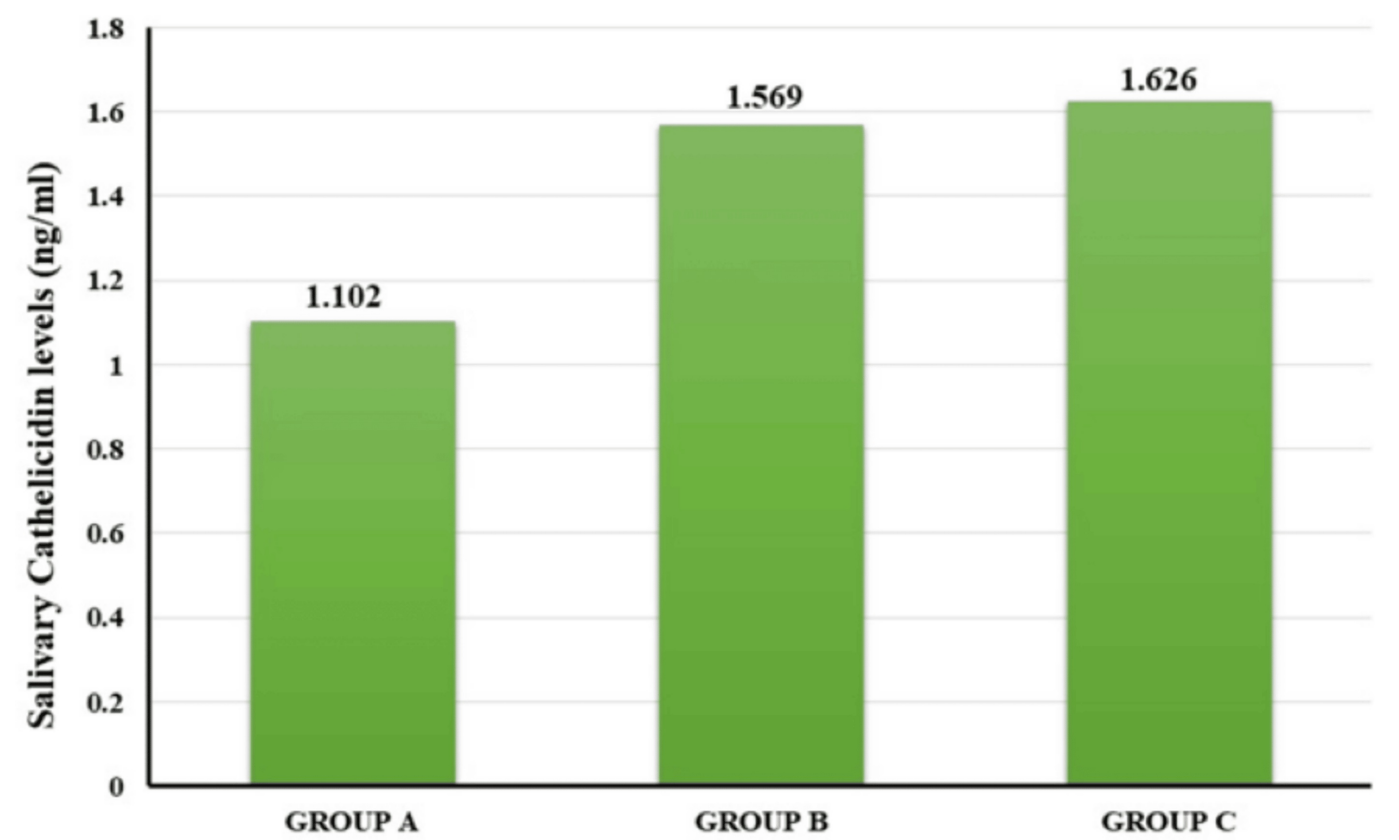
Mean values of salivary cathelicidin levels of Groups A (healthy), B (periodontitis patients without smokeless tobacco habit), and C (periodontitis patients with smokeless tobacco habit).

Clinical examination of the patients revealed that patients in Groups B and C showed poor oral hygiene, increased PPD, and higher clinical attachment loss. Clinical as well as biomolecular evaluation showed higher levels of periodontal parameters in Group C as compared to Group B. The intergroup comparison of periodontal parameters such as oral hygiene index-simplified, Russell’s periodontal index, PPD, clinical attachment level (CAL), and salivary cathelicidin and 8-isoprostane levels was done using Analysis of the variance formula test (ANOVA). Oral hygiene index values of Groups A, B, and C were 0.526, 2.397, and 3.613, respectively. Intergroup comparison showed a significant difference in all three groups (p=0.000). Regarding Russell’s index, the mean values of Groups A, B, and C were 0.139, 2.852, and 3.51, respectively. Intergroup comparison was statistically significant (p=0.000). The mean PPD of Group B was 6.45, and Group C showed a value of 7.42. The mean values of the CAL of Group B showed 3.81, and Group C showed 3.97. Intergroup comparison showed a significant difference in all three groups (p=0.000). The mean cathelicidin levels of Groups A, B, and C are 1.102, 1.569, and 1.626, respectively (Figure 2).

**Figure 3:**
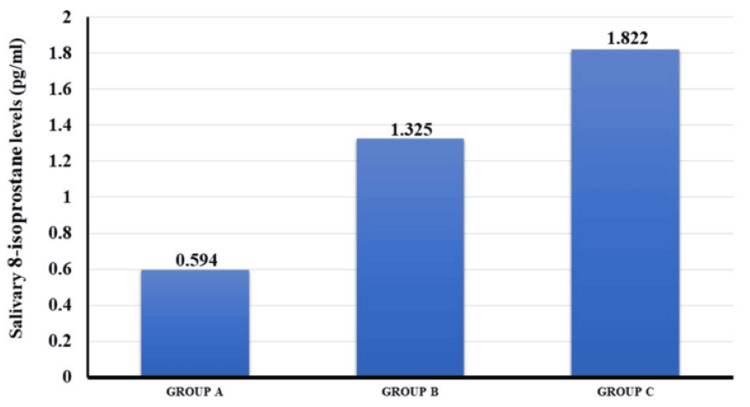
Mean values of salivary 8-isoprostane levels of Groups A (healthy), B (periodontitis patients without smokeless tobacco habit), and C (periodontitis patients with smokeless tobacco habit).

Intergroup comparison was statistically significant (p=0.000). The mean value of 8-isoprostane levels of Groups A, B, and C were 0.594, 1.325, and, 1.822, respectively (Figure 3).

The intergroup comparison revealed significant differences in all three groups (p=0.000) (Table 1).

**Table 1:** Intergroup comparison between oral hygiene index simplified, Russell’s index, probing pocket depth, clinical attachment level, cathelicidin levels, and 8-isoprostane levels using the ANOVA test. * p-value < 0.05: Level of statistical significance

Parameters	Groups	Mean	Standard Deviation	Standard Error	95% Confidence Interval for Mean		Minimum	Maximum	p-value
					Lower Bound	Upper Bound			
Oral Hygiene Index Simplified	Group A	0.526	0.2190	0.0393	0.445	0.606	0.1	0.8	0.000*
	Group B	2.397	0.4223	0.0758	2.242	2.552	1.5	3.2	
	Group C	3.613	0.4731	0.0850	3.439	3.786	2.9	5.2	
Russel’s index	Group A	0.139	0.0667	0.0120	0.114	0.163	0.0	0.2	0.000*
	Group B	2.852	0.6303	0.1132	2.620	3.083	1.8	4.0	
	Group C	3.510	0.3487	0.0626	3.382	3.638	2.7	4.2	
Probing Pocket Depth	Group A	NA	NA	NA	NA	NA	NA	NA	0.000*
	Group B	6.45	0.925	0.166	6.11	6.79	5	8	
	Group C	7.42	0.807	0.145	7.12	7.72	6	9	
Clinical Attachment Level	Group A	NA	NA	NA	NA	NA	NA	NA	0.000*
	Group B	3.81	1.195	0.215	3.37	4.24	0	6	
	Group C	3.97	0.875	0.157	3.65	4.29	3	6	
Cathelicidin	Group A	1.102	0.423	0.076	0.946	1.257	0.228	1.964	0.000*
	Group B	1.569	0.273	0.049	1.468	1.669	1.146	2.296	
	Group C	1.626	0.382	0.068	1.486	1.766	1.167	2.500	
8-isoprostane	Group A	0.594	0.203	0.036	0.519	0.668	0.228	0.983	0.000*
	Group B	1.325	0.136	0.025	1.274	1.376	1.022	1.582	
	Group C	1.822	0.260	0.046	1.727	1.917	1.278	2.411	

As all the parameters were highly significant in intergroup comparison, Tukey’s post-hoc test followed an analysis of the variance formula test (ANOVA). The group-wise analysis of all the periodontal parameters with salivary levels of cathelicidin and 8-isoprostane was performed. On the comparison of the OHIS, Russell’s index, PPD, and, CAL, the scores were the highest in Group C, followed by Groups B and A, respectively. It is statistically significant, suggesting that, with an increase in disease severity, the values of periodontal disease parameters are increasing. Cathelicidin (LL 37) levels in Group A are significantly lower than Group B (-0.4670) and C (-0.5240, p=0.813). No significant difference was found between Groups B and C (p=0.813). Lastly, for 8-isoprostane levels, Group A had significantly lower levels than Groups B (-0.7310) and C (-1.2280) (p=0.000). Group B had higher levels than Group A but lower than Group C (0.4960, p=0.000) (Table 2).

**Table 2:** Intergroup comparison between oral hygiene index simplified, Russell’s index, probing pocket depth, clinical attachment level, cathelicidin levels, and 8-isoprostane levels using Tuckey’s post-hoc test. * Statistically significant

Dependent Variable	(I) Group	(J) Group	Mean Difference (I-J)	Standard Error	P-value	95% Confidence Interval	
						Lower Bound	Upper Bound
Oral Hygiene Index Simplified	Group A	Group B	-1.8710^*^	0.0984	0.000*	-2.105	-1.636
		Group C	-3.0871^*^	0.0984	0.000*	-3.322	-2.853
	Group B	Group A	1.8710^*^	0.0984	0.000*	1.636	2.105
		Group C	-1.2161^*^	0.0984	0.000*	-1.451	-0.982
	Group C	Group A	3.0871^*^	0.0984	0.000*	2.853	3.322
		Group B	1.2161^*^	0.0984	0.000*	0.982	1.451
Russel’s index	Group A	Group B	-2.7129^*^	0.1061	0.000*	-2.966	-2.460
		Group C	-3.3710^*^	0.1061	0.000*	-3.624	-3.118
	Group B	Group A	2.7129^*^	0.1061	0.000*	2.460	2.966
		Group C	-0.6581^*^	0.1061	0.000*	-0.911	-0.405
	Group C	Group A	3.3710^*^	0.1061	0.000*	3.118	3.624
		Group B	0.6581^*^	0.1061	0.000*	0.405	0.911
Probing Pocket Depth	Group A	Group B	-6.452^*^	0.180	0.000*	-6.88	-6.02
		Group C	-7.419^*^	0.180	0.000*	-7.85	-6.99
	Group B	Group A	6.452^*^	0.180	0.000*	6.02	6.88
		Group C	-0.968^*^	0.180	0.000*	-1.40	-0.54
	Group C	Group A	7.419^*^	0.180	0.000*	6.99	7.85
		Group B	0.968^*^	0.180	0.000*	0.54	1.40
Clinical Attachment Level	Group A	Group B	-3.806^*^	0.217	0.000*	-4.32	-3.29
		Group C	-3.968^*^	0.217	0.000*	-4.49	-3.45
	Group B	Group A	3.806^*^	0.217	0.000*	3.29	4.32
		Group C	-0.161	0.217	0.739	-0.68	0.36
	Group C	Group A	3.968^*^	0.217	0.000*	3.45	4.49
		Group B	0.161	0.217	0.739	-0.36	0.68
Cathelicidin	Group A	Group B	-0.467	0.092	0.000*	-0.688	-0.246
		Group C	-0.524	0.092	0.000*	-0.745	-0.303
	Group B	Group A	0.467	0.092	0.000*	0.246	0.688
		Group C	-0.057	0.092	0.813	-0.278	0.164
	Group C	Group A	0.524	0.092	0.000*	0.303	0.745
		Group B	0.057	0.092	0.813	-0.164	0.278
8-isoprostane	Group A	Group B	-0.731	0.052	0.000*	-0.857	-0.605
		Group C	-1.228	0.052	0.000*	-1.353	-1.103
	Group B	Group A	0.731	0.052	0.000*	0.605	0.857
		Group C	-0.496	0.052	0.000*	-0.623	-0.370
	Group C	Group A	1.228	0.052	0.000*	1.103	1.353
		Group B	0.496	0.052	0.000*	0.370	0.623

Periodontal parameters, such as oral hygiene index, Russell’s index, PPD, and CAL, were correlated with levels of cathelicidin and 8-isoprostane using Pearson’s coefficient for correlation. The correlation coefficients were significant at the 0.01 level. Higher values of periodontal parameters were positively correlated with high salivary cathelicidin and 8-isoprostane levels. This implies that, as periodontal parameters increase, there is a corresponding increase in cathelicidin and 8-isoprostane levels. The higher values of clinical parameters indicate higher periodontal destruction and are in accordance with high levels of cathelicidin and 8-isoprostane (Table 3).

**Table 3:** Pearson’s correlation between oral hygiene index simplified, Russell’s index, probing pocket depth, clinical attachment level, cathelicidin levels, and 8-isoprostane levels. ** Correlation is significant at the 0.01 level.

		Oral Hygiene Index Simplified	Russel’s index	Probing Pocket Depth	Clinical Attachment Level	LL 37	8-isoprostane
Oral Hygiene Index Simplified	Pearson Correlation	1	0.943^**^	0.913^**^	0.824^**^	0.551^**^	0.900^**^
	p-value	NA	0.000	0.000	0.000	0.000	0.000
Russel’s index	Pearson Correlation	0.943^**^	1	0.948^**^	0.877^**^	0.496^**^	0.882^**^
	p-value	0.000	NA	0.000	0.000	0.000	0.000
Probing Pocket Depth	Pearson Correlation	0.913^**^	0.948^**^	1	0.874^**^	0.572^**^	0.877^**^
	p-value	0.000	0.000	NA	0.000	0.000	0.000
Clinical Attachment Level	Pearson Correlation	0.824^**^	0.877^**^	0.874^**^	1	0.475^**^	0.798^**^
	p-value	0.000	0.000	0.000	NA	0.000	0.000
Cathelicidin	Pearson Correlation	0.551^**^	0.496^**^	0.572^**^	0.475^**^	1	0.510^**^
	p-value	0.000	0.000	0.000	0.000	NA	0.000
8-isoprostane	Pearson Correlation	0.900^**^	0.882^**^	0.877^**^	0.798^**^	0.510^**^	1
	p-value	0.000	0.000	0.000	0.000	0.000	NA

## Discussion

Periodontitis is a multifactorial disease marked by persistent inflammation of the periodontium, leading to the gradual destruction of periodontal structures [9]. Pathogenic microorganisms initiate the disease by invading periodontal tissues and triggering the host's inflammatory response, activating an inflammatory cascade involving various biomolecules [10]. Chemical mediators such as cytokines, leukotrienes, and prostaglandins regulate this response, restoring homeostasis in the periodontium [11]. Cathelicidin and 8-isoprostane are such biomolecules that are present in the present during inflammation.

Tobacco use exacerbates periodontal disease by increasing oxidative stress and altering immune responses, leading to microvascular dysfunction and higher complication risks [12]. Regardless of form, tobacco exposure affects salivary biomolecule concentrations and inflammatory responses in the periodontium, impacting disease progression and therapy outcome. Saliva contains these inflammatory molecules, genetic material, biomolecules, etc., which are used as a diagnostic tool. This study evaluates the influence of smokeless tobacco on salivary cathelicidin LL-37 and 8-isoprostane levels in periodontal health and disease. Individual studies in the past have compared the effect of tobacco products on either cathelicidin (LL 37) or 8-isoprostane respectively. To the best of our knowledge, there are no studies that comparatively evaluate the relationship between salivary markers such as 8-isoprostane and cathelicidin in periodontal health and disease with the effect of smokeless tobacco.

In the present study, Group C had the oldest participants, with a higher mean age than Groups A and B, which showed higher periodontal destruction. Clark et al. explained a similar relationship between age and periodontal destruction [13]. The reason for severe periodontal destruction between older age groups can be the inflammatory changes due to changes in immune response contributing to periodontal disease severity.

In the present study, a higher number of male participants were consuming smokeless tobacco. Angelov et al. showed a contrasting trend with more female participants and a smaller number of male participants involved in smokeless tobacco use [14]. The reason behind more male smokeless tobacco users is that male participants were from rural areas with poor oral health awareness and educational background. The reason for the contrast with our study could be due to disproportionate gender inclusion in the study.

In the present study, the OHIS was measured. Group C showed significantly higher values than Group A. Katuri et al. [15] and Verma et al. [16] showed similar results wherein the group of participants with smokeless tobacco use showed higher mean scores on the OHIS. The smokeless tobacco habit and periodontitis can indicate poor oral hygiene, which may be the result of combined localized contact and direct absorption of chemicals from tobacco, leading to severe tissue destruction.

In this study, Russell’s periodontal index was measured to assess the periodontal health status of study subjects. In the present study, the impact of smokeless tobacco use was correlated with higher periodontal destruction. Bangera et al. confirmed that Russell’s periodontal index is a valuable periodontal index in assessing periodontal disease severity [17]. The chronic habit of smokeless tobacco is correlated with increased periodontal destruction.

In the study, higher PPD values in Group C indicate that smokeless tobacco is a risk factor for periodontal disease. Mittal et al. reported similar results with mean PPD (4.25 ± 0.53) in smokeless tobacco users [18]. A meta-analysis by Pesce et al. observed that an increase in pocket depth was associated with tobacco use [19]. The higher PPD can be a result of high pH and nicotine content, which is absorbed through oral mucosa rapidly making the population more susceptible to higher probing pocket depths.

In the present study, periodontal destruction was also assessed. Intergroup comparison showed a significant difference in all three groups (p=0.000), with a higher clinical attachment level in Group C. Bhandarkar et al. and Goel et al. showed similar results and reported that tobacco induces localized tissue damage associated with periodontal destruction [20,21].

In the present study, the highest mean value of cathelicidin (LL 37) was observed in Group C. Overall, there was scanty literature available to explain the relationship between smokeless tobacco and cathelicidin in relationship with periodontal health and disease. In the present study, elevated levels of LL 37 were observed with periodontitis and environmental factors of the smokeless tobacco habit. The contrasting results were observed by Kotecha et al. where low levels of LL 37 were correlated with severe periodontitis [22]. The reason behind the contrast could be the immunosuppressive effect of smokeless tobacco and the state of disease activity.

The present study also evaluated oxidative stress markers (i.e., 8-isoprostane in all three study groups). Intergroup comparison was statistically significant. Kurita et al. and Erve et al. showed similar results with elevated 8-isoprostane levels in smokers [23,24].

In the present study, Pearson’s correlation of OHIS and Russell’s index showed strong positive correlations with the increased amount of PPD, CAL, cathelicidin, and 8-isoprostane levels. It also proved that tobacco product-induced oxidative stress increases periodontal tissue damage. This result was in accordance with Dong et al., who highlighted the interrelationship between oxidative stress marker 8-isoprostane and inflammatory biomarker cathelicidin [25]. The levels of cathelicidin and 8-isoprostane biomarkers were influenced by smokeless tobacco. The reason could be the potential mechanism of the cathelicidin-related antimicrobial peptide (CRAMP) cascade. CRAMP regulates reactive oxygen species (ROS), and CRAMP regulates oxidative stress by controlling neutrophil function and cell differentiation. Therefore, a positive correlation was observed between tobacco use, cathelicidin, and 8-isoprostane levels with higher periodontal tissue destruction.

There were some drawbacks of this study; for example, a limited sample size made it difficult to extrapolate the result. The examiner was not blinded to avoid observer bias. Other confounding factors should have been included in the study, such as obesity, physical status, etc. The study's scope was also restricted to specific populations or demographics, potentially limiting the generalizability of the results.

## Conclusions

In the present study, individuals with periodontitis and a smokeless tobacco habit exhibited severe periodontal destruction as compared to healthy individuals. Similarly, patients with a smokeless tobacco habit exhibited higher inflammation and oxidative stress as evident with significantly higher levels of salivary cathelicidin and 8-isoprostane compared to healthy individuals. Individuals with smokeless tobacco habit presented with severe periodontal destruction and higher oxidative stress. This study found a unique positive correlation between the inflammatory marker cathelicidin and the oxidative stress marker 8-isoprostane. Further multicentred research with diverse racial and population groups is needed to validate these findings and ensure broader applicability. The study highlights the effects of smokeless tobacco on periodontal health and disease and explores the potential diagnostic and therapeutic implications of salivary cathelicidin and 8-isoprostane. Future studies should aim for a more comprehensive and generalizable understanding to enhance the reliability and applicability of these findings.
